# New insights on basivenal sclerites using 3D tools and homology of wing veins in Odonatoptera (Insecta)

**DOI:** 10.1038/s41598-017-18615-0

**Published:** 2018-01-10

**Authors:** Lauriane Jacquelin, Laure Desutter-Grandcolas, Ioana Chintauan-Marquier, Renaud Boistel, Daran Zheng, Jakub Prokop, André Nel

**Affiliations:** 1 Institut de Systématique, Évolution, Biodiversité, ISYEB - UMR 7205 – CNRS, MNHN, UPMC, EPHE, Muséum national d’Histoire naturelle, Sorbonne Universités, 57 rue Cuvier, CP 50, Entomologie, F-75005 Paris, France; 2 Université de Poitiers - UFR SFA, iPHEP UMR CNRS 7262, Bât B35 - TSA 51106, 6 rue Michel Brunet, F-86073 Poitiers, Cedex 9 France; 3 State Key Laboratory of Palaeobiology and Stratigraphy, Nanjing Institute of Geology and Palaeontology, Chinese Academy of Sciences, 39 East Beijing Road, Nanjing, 210008 China; 4 Department of Zoology, Charles University, Viničná 7, CZ-128 43 Praha 2, Czech Republic

## Abstract

Being implied in flight, mimetism, communication, and protection, the insect wings were crucial organs for the mega diversification of this clade. Despite several attempts, the problem of wing evolution remains unresolved because the basal parts of the veins essential for vein identification are hidden in the basivenal sclerites. The homologies between wing characters thus cannot be accurately verified, while they are of primary importance to solve long-standing problems, such as the monophyly of the Palaeoptera, viz. Odonatoptera, Panephemeroptera, and Palaeozoic Palaeodictyopterida mainly known by their wings. Hitherto the tools to homologize venation were suffering several cases of exceptions, rendering them unreliable. Here we reconstruct the odonatopteran venation using fossils and a new 3D imaging tool, resulting congruent with the concept of Riek and Kukalová-Peck, with important novelties, viz. median anterior vein fused to radius and radius posterior nearly as convex as radius anterior (putative synapomorphies of Odonatoptera); subcostal anterior (ScA) fused to costal vein and most basal primary antenodal crossvein being a modified posterior branch of ScA (putative synapomorphies of Palaeoptera). These findings may reveal critical for future analyses of the relationships between fossil and extant Palaeoptera, helping to solve the evolutionary history of the insects as a whole.

## Introduction

Despite recent advances, the monophyly of the group Palaeoptera remains controversial, mainly because of the antiquity of this group rendering the molecular analyses uncertain^1–3^, and the fact that one of its major components, the Palaeodictyopterida are strictly fossil and mainly known by the wings^4^. Odonatoptera have a highly complicate and derived venation, showing numerous fusions of the main veins. Although there is a rather broad consensus today for the use of Riek and Kukalová-Peck’s odonatopteran wing venation nomenclature and homology^5^, Nel *et al*.^6^ showed that the venation of the Mesozoic Isophlebiidae cannot be explained by the hypotheses of previous authors (history of these proposals^7^). As no examination of the hidden extreme bases of the main veins has ever been made to verify the validity of Riek and Kukalová-Peck’s hypothesis, the identities of the veins have in fact never been checked. Some issues are crucial to define homologous characters with the other Palaeoptera, viz. the exact course of the median anterior vein (MA)^6^; the existence of a radius posterior (RP) nearly as convex as radius anterior (RA)^8^; the nature of the most basal primary antenodal crossvein (Ax0) as a modified posterior branch of the subcostal anterior (ScA)^5,9^; the existence of a ScA fused to costal vein (C); and the precostal vein (PC) alleged to be present after Riek and Kukalová-Peck^5^. Also Nel *et al*.^6^ considered that MA is fused to radius (R) while Riek and Kukalová-Peck^5^ supposed that there is long stem of M divided at the level of the arculus into MA and median posterior (MP).

Fossil wings are generally too poorly preserved to solve these problems that concern structures of the extreme base of the wings. However, there are few exceptions like three dimensional state of preservation in siderite nodules, allowing to study the wing joints with veinal basivenales rarely documented on Carboniferous insects. Such examples are known like a palaeodictyopteran *Mazonopterum wolfforum* Kukalová-Peck and Richardson, 1983, or a typically neopteran architecture of basivenal sclerites demonstrated on Paoliida (stem Dictyoptera)^10,11^. Similar problems exist for extant taxa, especially in Odonata in which the vein bases are hardly visible, hidden in the fused, sclerotized structures of the wing base. The basivenal sclerites have been studied only from outside^12^. Clarifying the odonatopteran venation is the necessary first step to solve the relationships within the fossil palaeopteran groups.

Here we had the opportunity to analyze an exquisitely preserved basal half of a wing of a Triassic Odonatoptera Triadophlebiomorpha (*Zygophlebia tongchuanensis* Zheng *et al*., 2017) showing the structures of the extreme wing base. We also made the 3D CT-scan reconstruction of the extreme base of the forewing of an extant *Aeshna isoceles* Müller, 1767. The method was successfully developed by our team to study the venation of the Orthoptera Ensifera^13^ and Phasmatodea (in prep.).

## Forewing basal structures of an extant ***Aeshna***

**Figure 1:**
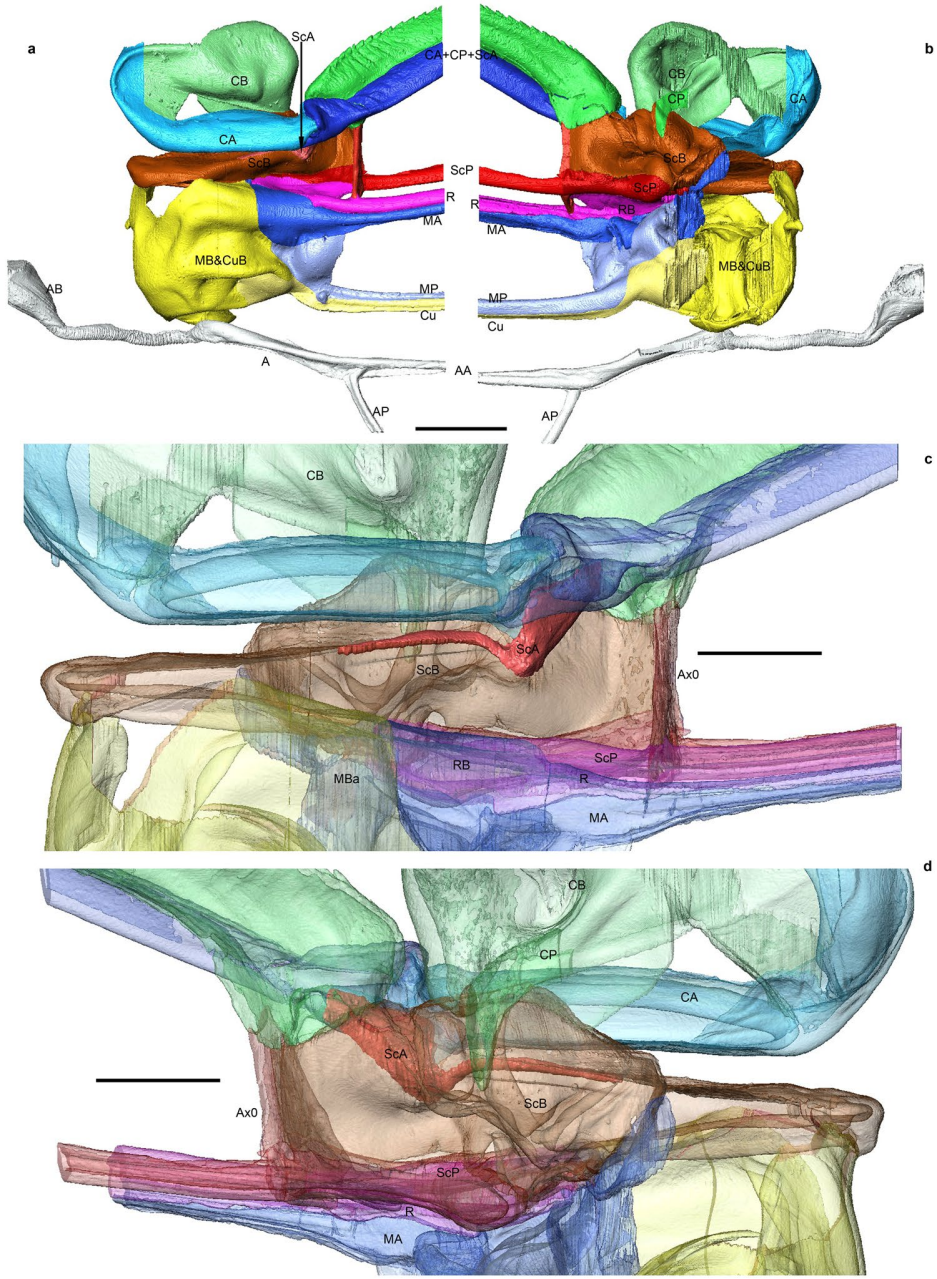
Wing venation and basivenales of *Aeshna isosceles*. (**a)** 3D modeling of wing venation using XMT, view from above. **(b)** 3D modeling of wing venation using XMT, view from below. **(c)** Transparent magnification of anterior part, view from above. **(d)** Transparent magnification of anterior part, view from below. Scale bars = 1 mm (copyright L.J.).

**Figure 2:**
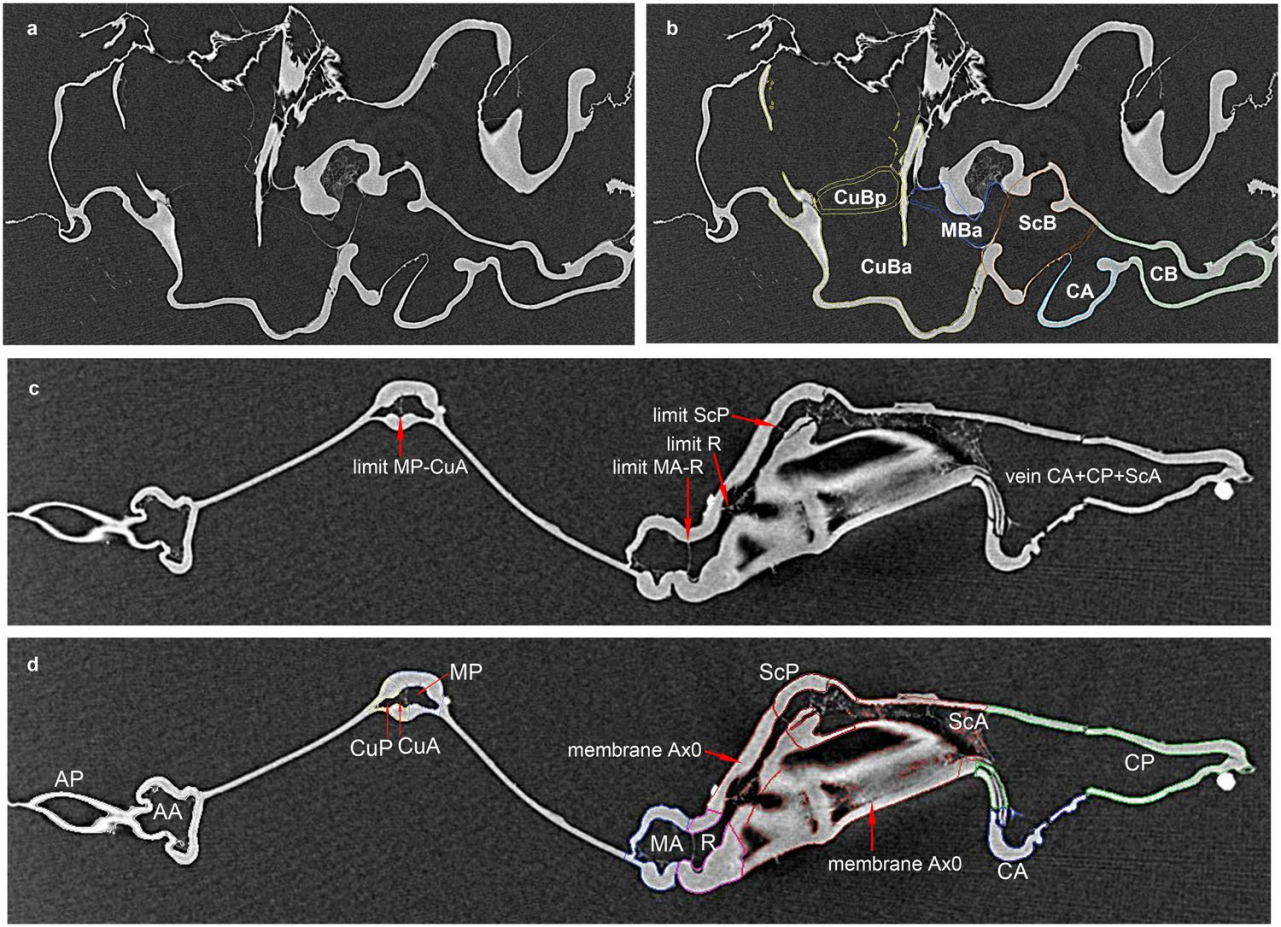
Wing venation and basivenales of *Aeshna isosceles*. **(a**,**b)** cut at level of base of median basivenale MBa (a not colored; b colored). **(c**,**d)** cut at level of Ax0 (c not colored; d colored) (copyright L.J.).

(Figures 1 and 2, Suppl. movies 1–17; Suppl. Data for the code of colors of veins and basivenales). There is no trace of a jugal vein or a jugal basivenale; anal vein is emerging from basivenale AB situated in a much more basal position than the other basivenales; it is divided distally into AA and AP. The veins MA, MP, and Cu emerge from what looks from the outside as a large shared basivenale. However this structure is subdivided internally into three sub-basivenales by weaker membranes (Fig. 2a), one for MA (MBa), one for MP (MBp) and two for Cu (CuBa and CuBp) (Suppl. movies 12–17); the MA’s and the MP’s are basally fused together into a MB. MA is clearly going into the same vein as R, but their basivenales are independent; the composite vein RA + RP + MA contains the three veins separately in its basal part near the transverse vein Ax0, but these three veins are distally fused into a unique vein, before RP + MA separates again into the arculus. ScP is emerging from a basivenale ScB independent of RB, and the so-called ‘costal margin’ results from the fusion of three different veins, viz. ScA emerging at the very base of ScB and appressed to it for a long distance (Fig. 1c,d, Suppl. movies 7–9), a concave vein CP, and a convex vein CA that is in an anterior position relatively to CP (Suppl. movies 2–5); CA has a long course between ScB and CB, makes an anterior curve and ends into basal part of CB; CP emerges from the mid part of the posterior side of CB. Ax0 has a complex origin, apparently with a branch of ScA going into it (Fig. 1c, Suppl. movie 10).

## Forewing basal structures of the fossil ***Zygophlebia tongchuanensis***

**Figure 3:**
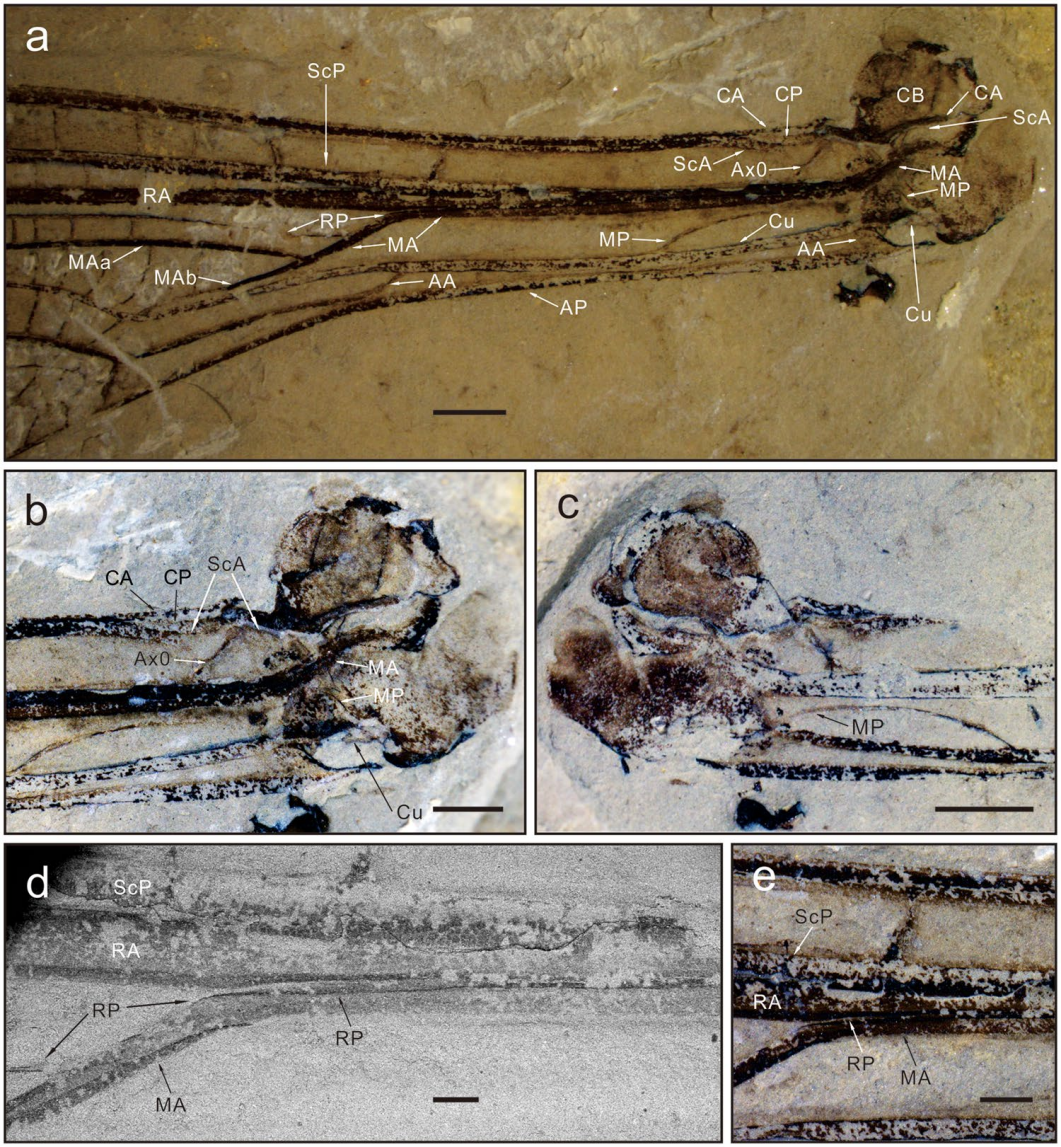
Wing venation of *Zygophlebia tongchuanensis*. **(a)** Wing base. **(b**,**c)** Magnification of extreme base (b part; c counterpart). **(c**,**d)** Magnification of arculus. Scale bars = 200 µm. (copyright D.Z.).

The pattern of veins and basivenales of the fossil *Zygophlebia tongchuanensis* is nearly identical to that of the extant *Aeshna*, except for AA going into Cu at wing base (wing petiolation), Ax0 much more oblique and with the posterior fork of ScA well visible (Fig. 3).

## Discussion

We combine the precise observations of basal wing structures in an extant taxon studied with 3D tomography with the study of a well-preserved fossil. We are thus in a position to propose, for the first time, a consensual interpretation of the venation of the Odonatoptera to be compared to other Palaeoptera, identifying individually both the basivenal sclerites and the principal veins of the wing.

### Basivenal sclerites

All the basivenales, except the jugal one, are clearly present. Kukalová-Peck^14^ indicated the presence of jugal vein in *Protereisma* sp. (currently in Ephemeroptera). Kukalová-Peck^15^ interpreted the presence of jugal veins in prothoracic winglets of Palaeodictyoptera and Odonatoptera: Geroptera, while our aeshnid wing shows no trace of a jugal vein or a jugal basivenale. The confirmation of the existence of a jugal vein or basivenale needs further investigation in other extant insects, using the same technical tools of X-ray tomography.

The CuB and MB are apparently fused when seen from outside (corresponding to the axillary plate^12^), but they remain well separated by weak membranes, also visible outside by sutures. The extant *Aeshna isosceles* (Müller, 1767), *Zygophlebia tongchuanensis*, the Carboniferous Meganeuridae^16^, and the basal Odonatoptera *Kirchnerala* Petrulevičius and Gutiérrez, 2016 have very large basivenales for Costa and for M&Cu while those of Sc and R are narrower^8^. Thus these characters are probably present in all Odonatoptera. It will be necessary to verify if they are proper to this clade or present in some other palaeopteran groups. In the paleodictyopteran nymphs like *Idoptilus onisciformis* Wootton, 1972, the future basivenales seem to be clearly divided into basisubcostale and axillary plate^17^. In extant Ephemeroptera, Cu seems to emerge from a transverse basivenale separated from that of M^18,19^, but this needs to be confirmed by the examination of the internal structures of these sclerites.

### Median vein

We confirm the composite nature of the vein ‘M + Cu’ sensu^5^, with an important correction: this vein is in fact MP + Cu^6^. The vein MA is not fused with Cu: it runs into the radial vein, and separates from RA together with RP into the arculus (Fig. 1, Suppl. movies 12–14). This pattern of venation is congruent with the numerous cases of Odonatoptera in which there is no posterior side of the arculus; while under the hypothesis of Riek & Kukalová-Peck, MA is basally fused with MP and Cu and makes a ‘gap’ (missing part) to go into the arculus and re-emerge immediately distally. Bechly^20^, following^5^, supposed that, in the Neodonatoptera, ‘the base of MA has lost its connection with the medial stem and is secondarily fused with RP’. This sentence is ambiguous, in the sense that Bechly did not indicate where the base of MA is: at wing base or only in the arculus together with RP?

The hypothesis of Riek & Kukalová-Peck^5^ is based on their interpretation of the pattern of venation of the ‘basal’ Odonatoptera *Eugeropterum lanatum* Riek, 1984, in which M is separated from R and Cu and divided into MA and MP in a very distal position. Kukalová-Peck^21^ gave a photograph of the wing base of this taxon in which the vein ‘RP’ basal of the arculus is neutral compared to the strongly convex RA and to the strongly concave RP distal of the arculus; basal ‘M’ is also neutral compared to the concave Cu and the strongly convex distal MA. Also the short vein in the arculus between ‘RP’ and distal part of MA is very strong, stronger than the alleged base of MA from the stem of ‘M’. These facts are better congruent with our hypothesis that the alleged vein ‘M’ is simply the neutral MP ending into Cu and the alleged vein ‘RP’ is the neutral RP + MA, from which the concave RP and the convex MA emerge distally in the arculus, as in all other Odonatoptera (see below).

In the Liassic Heterophlebiomorpha *Paraheterophlebia marcusi* Nel & Henrotay, 1993, the extant *Epiophlebia superstes* (Selys, 1889), and the Triassic *Pseudotriassothemis okafujii* Fujiyama, 1991, the alleged vein ‘M’ (sensu^5^) is neutral, neither concave nor convex^6,22,23^. Pritykina^24^ described several Triadophlebiomorpha with an alleged convex MP ending into a concave Cu, but the very well preserved type wing of *Zygophlebia tongchuanensis* shows a neutral MP ending in the convex Cu, a double vein RP + MA that is divided into RP going to the RB and MA going into the large basivenale of M & Cu, even MP is emerging near the basal part of this basivenale, as in our extant *Aeshna* (see Fig. 3b).

In *Geropteron arcuatum* Riek, 1984, MA is fused with R and re-emerging with RP in the arculus as in modern Odonata. The vein MP (‘M’ sensu^5^) is less convex than RA and RP + MA and ends into Cu.

*Kirchnerala treintamil* Petrulevičius and Gutiérrez, 2016 clearly shows a neutral MP independent of R and Cu and a strongly convex RP + MA independent of convex RA^8^. RP + MA has the same diameter and convexity as the distal MA, while the distal RP is clearly concave and much narrower than RP + MA and MA.

In conclusion, in all the taxa with a MP and a Cu separated at their bases, MP is concave, neutral, or at least less convex that the vein RP + MA. This pattern is quite homogenous. We consider that the veins RP and MA are basally fused in all Odonatoptera as a putative synapomorphy, RP + MA is either neutral or as convex and as wide as the distal MA, while the distal RP is concave. The neutral or concave MP is fused with Cu, either at wing base or slightly more distally.

### Remark

The Jurassic *Tarsophlebiopsis mayi* Tillyard, 1923 (Tarsophlebiidae, putative sister group of Odonata) is supposed to have a particular fusion of a vein emerging from radius close to wing base and ending into the concave ‘Cu’, but after the photograph of the type specimen, the vein ‘Cu’ is partly destroyed between the wing base and the arculus, and the alleged strange vein probably does not exist^25^.

### Nature of the anterior wing margin

Riek and Kukalová-Peck^5^ interpreted the anterior wing margin of Odonatoptera as the fusion of a convex PC&CA, a concave CP and a convex ScA. The present study confirms the existence of a ScA ending in the anterior wing margin in the extant Odonata, as in the most basal Odonatoptera. We can also confirm the presence of two other veins, one concave and one convex but at this time we could not observe any trace of a vein PC, but only one large basivenale CB (corresponding to the ‘proximal costal plate’ sensu^12^), from which the two veins CA and CP emerge. The ‘distal costal plate’ sensu^12^ corresponds in fact to the re-emergence of CP and ScA that go into the anterior wing margin.

In our *Aeshna* sp., a hidden posterior branch of ScA seems to go into the most basal primary antenodal crossvein Ax0. Also in *Zygophlebia tongchuanensis*, Ax0 is clearly oblique and corresponds to a posterior branch of ScA. These results are in accordance with the very precise previous observations of Bechly^20^. This situation is similar to what happen for the Permian mayflies *Protereisma* or *Alexandrinia* in which an oblique transverse basal vein is similar to Ax0 and could correspond to a posterior fork of ScA^5,9^.

Basally separated and convex ScA with maybe a posterior branch forming a crossvein between anterior margin and ScP also occurs in some members of the palaeodictyopterid Megasecoptera, viz. *Eubrodia dabasinskasi* Carpenter, 1967 or *Brodia priscotincta* Scudder, 1881 (Brodiidae)^26^. This group characterized by remarkably petiolate wings is considered as a close relative of Palaeodictyoptera due to a specialized type of mouthparts in form of a rostrum as synapomorphy for all Paleodictyopterida.

This special shape of ScA is a putative synapomorphy of Odonatoptera with Ephemeroptera, and (at least some) Palaeodictyopterida, to be confirmed by the reconstruction of an extant mayfly wing using the same CT-scan technique.

Another striking feature is the presence of well-separated concave CP discernable between ScA and anterior wing margin, which was omitted in the original description of *Zygophlebia* ^27^. Such basal organization of the veins CP and ScA distally forming the leading edge of the wing is also known in *Namuroningxia elegans* Prokop and Ren, 2007 (Palaeodictyoptera: Namuroningxiidae)^28^. The examination of latter species by ESEM uncovered the cuticular microstructures as knob like tubercles on these veins and simultaneously showed also the presence of a vein CA in the leading edge of the wing in this Palaeodictyoptera^29^. This character is also a putative synapomorphy of Odonatoptera with the Palaeodictyoptera, to be searched in the Ephemeroptera and the Neoptera too.

## Conclusion

The present study confirms the efficiency of the 3D imaging tool to observe the hidden structures at the wing bases of extant insects, i.e., check for the presence of basivenal sclerites and identify the veins. We could solve three long-standing problems concerning the venation of the Odonatoptera, viz. the structure of their basivenal sclerites, the exact nature of the median vein, and the exact nature of the ‘costal’ veins. As for the Ensifera^13^, the odonatopteran wing venation and the structures of the basivenal sclerites are a combination of very complex structures, not discernable using traditional light microscopy tools, and a potential source of new characters to be used in phylogenetic analyses. To achieve this scope, it will be necessary to reconstruct and analyze the wings of representatives of all other pterygote orders, in order to follow the modifications through time of homologous structures in extant and fossil insect wings, and thus solve a problem persisting for two centuries.

## Methods

### Materials

The studied specimen of *Zygophlebia tongchuanensis* (NIGP162226a-NIGP162226b) is housed at the Nanjing Institute of Geology and Palaeontology, Chinese Academy of Sciences.

### Imaging

The extant specimen of *Aeshna* was imaged under X-ray, with phase contrast, at the microtomograph of the University of Poitiers.

## Electronic supplementary material

Supplementary Information

Supplementary movie 1

Supplementary movie 2

Supplementary movie 3

Supplementary movie 4

Supplementary movie 5

Supplementary movie 6

Supplementary movie 7

Supplementary movie 8

Supplementary movie 9

Supplementary movie 10

Supplementary movie 11

Supplementary movie 12

Supplementary movie 13

Supplementary movie 14

Supplementary movie 15

Supplementary movie 16

Supplementary movie 17
