# Supplementary material for: New insights on basivenal sclerites using 3D tools and homology of wing veins in Odonatoptera (Insecta)
Source: Sci Rep. 2018 Jan 10;8:238. doi: 10.1038/s41598-017-18615-0 (PMC5762858; doi:10.1038/s41598-017-18615-0)
Supplement: Supplementary file 1 — Supplementary Information [file 41598_2017_18615_MOESM1_ESM.pdf]

**New insights on basivenal sclerites using 3D tools and homology of wing veins in dragonflies and griffenflies (Insecta: Odonatoptera)**

Lauriane Jacquelin, Laure Desutter-Grandcolas, Ioana Chintauan-Marquier, Renaud Boistel, Daran Zheng, Jakub Prokop, & André Nel

Supplementary Data

Material and method

The type specimen of *Zygophlebia tongchuanensis* (NIGP162226a-NIGP162226b, Nanjing Institute of Geology and Palaeontology, Chinese Academy of Sciences) was photographied under a high resolution, under alcohol, using a Zeiss Stereo Discovery V16 microscope system, and analyzed under a scanning electron microscope (SEM-EDS) at variable voltages. Micro-surface information was obtained under the low vacuum mode (100 Pa in sample chamber) of the LEO1530VP SEM with the accelerating voltage 5–20 kV. The SEM analyses were performed in the State Key Laboratory of Palaeobiology and Stratigraphy, Nanjing Institute of Geology and Palaeontology, University of Chinese Academy of Sciences. We also re-examine the wing bases of two Carboniferous Meganeuridae (*Meganeura monyi* (Brongniart, 1884), specimens R. 52938 and R. 52938, MNHN coll.).

The forewing base with the attached sclerites of the specimen of the extant *Aeshna isoceles* Müller, 1767 was cut, put in a plastic tube, and imaged under X-ray, with phase contrast, at the microtomograph of the University of Poitiers, according to the protocols reported in the literature[1,2,3]. We used a microtomograph RX solutions EasyTom XL Duo, using a Hamamatsu nanofocus160 kV 8W, source and a Varian-Paxscan 2520DX CsI detector. Imaging was done at the Centre for Microtomography of the University of Poitiers (France).

Scans were performed at 70 kV and 33 μA. The geometry was set to obtain a 3.45103 μm voxel size in the reconstructed three-dimensional images. For the reconstruction we used the FDK algorithms of Xact. 4.3. The dataset consists of 1184 projections taken over 360° for the wings of the specimen, with 0.5 s of exposure time for each projection.

Slices are windowed to obtain the best contrast in soft tissues and reduce the grey level values to 8-bits, i.e., to reduce the data size, and finally create a ‘slice’, an orderly stack of tomograms to an easy handling thereafter. For this scope, we used the ImageJ® (64-bit) software (Rasband, W.S., ImageJ, U.S. National Institutes of Health, Bethesda, Maryland, USA, http://imagej.nih.gov/ij/, 1997-2015).

3D volume rendering (Isosurface) was used to visualize the sub-set of selected voxels of the wings structure in AVIZO (FEI, Merignac, France, https://www.fei.com/software/amira-avizo/). This was performed using a manual segmentation tools (the paint brush) with a selection of threshold of level of grey.

Color used for veins and sclerites

A specific color was assigned for each homologous structure, viz., pale green for CB, dark green for CP, pale blue for basal part of CA, very dark blue for CA+ScA, dark orange for ScA, dark red for ScB, red ScP, dark pink for RB and R, dark blue for MA, pale blue for MP, pale yellow for Cu, yellow for MB+CuB; pale grey for anal veins and AB. The segmentation process resulted for each dataset in a ‘label’ dataset with the same dimensions as the corresponding slice. The microtomography analyses show the veins and thoracic basal sclerites. In order to verify the 3D modeling, the specimen was observed under a binocular microscope, model Olympus SZX9, with a target PLAPO 1X DF-2.

Definitions

**Vein**: hollow tubular structure formed by the coupling of the upper and lower sheets of the wing membrane, supporting and consolidating it. The most important veins are longitudinal and can contain nerve, trachea, and hemolymph. The smaller veins, called crossveins, are transverse, stiffening structures. Veins can be thick or thin, broadened or narrowed, with an oval or subquadrangular section[4], corrugated or hollow[5]. Veins are named and studied from the most posterior to the most anterior.

Abbreviations: Independently of the problems of homology, the names and abbreviations are as follows: J = jugal vein; A = anal vein; Cu = cubital vein; M = median vein; R = radial vein; Sc = subcostal vein; C = costal vein; PC = precostal vein; XA = anterior branch of a vein X; XP posterior branch of a vein X.

**Basivenal sclerite**s (herein named basivenales): sclerotized blood cavities, at the wing base, by which the hemolymph arrives in the wing[6]. The basivenal sclerites are the most distal series of sclerites between the thorax and the wing[6,7]. They are named: JB jugal basivenale; AB anal basivenale; CuB cubital basivenale; MB median basivenale; RB radial basivenale; ScB subcostal basivenale; and CB costal basivenale. The existence of this complete series in all insect orders has to be verified.

**Trachea**: tube of the respiratory system that can ramify into tracheoles, to bring the air to all the organs. Riek and Kukalová-Peck[8] proposed an interpretation of the tracheae of an extant Anisoptera (*Tramea* sp.), which is very hard to interpret because it is two dimensional. The drawing does not show several important structures at the wing base.

**Vein concavity/convexity**: a concave vein (indicated by a '-' in figures) is placed in low position on the ventral sheet of the wing membrane, while a convex vein (indicated by a '+' in figures) is placed in high position on the dorsal sheet of the wing membrane. A vein between the two sheets is neutral[4]. The convexity of a vein can vary in its path (the most spectacular cases concern the vein that bears the file in crickets[9].

**Wing**: flight body composed of two layers of epidermal cells forming upper and lower layers. It can be thin, membranous or thick, discolored, transparent, or brightly colored, covered with setae or scales. The wing is traversed by an array of ribs and tracheae[4].

References


1. Boistel, R. *et al*. Shake rattle and roll: the bony labyrinth and aerial descent in squamates. *Integr. Comp. Biol.* **56**, 957–968 (2011).
2. Lauridsen, H. *et al*. Inside out: modern imaging techniques to reveal animal anatomy. *PLoS ONE* **6**, e17879. (2011).
3. Zanette, I. *et al*. Looking inside marine organisms with magnetic resonance and X-ray imaging. In Imaging marine life (ed. EG Reynaud), pp. 122–184. Weinheim, Germany: Wiley-VCH Verlag GmbH & Co. KgaA. (2013).
4. Séguy, E. Introduction à l'étude morphologique de l'aile des insectes. *Mém. Mus. Natl Hist. Nat.*, Série A, *Zool.* **21**, 1–248 (1959).
5. Desutter-Grandcolas, L. Functional forewing morphology and stridulation in crickets (Orthoptera: Grylloidea). *J. Zool.* **236**, 243–252 (1995).
6. Kukalová-Peck, J. Origin and evolution of insect wings and their relation to metamorphosis, as documented by the fossil record. *J. Morphol.* **15**, 53–126 (1978).

7. Kukalová-Peck, J. Origin of the insect and wing articulation from the arthropodan leg. *Can. J. Zool.* **61**, 1618–1669 (1983).
8. Riek, E.F. & Kukalová-Peck, J. A new interpretation of dragonfly wing venation based upon Early Carboniferous fossils from Argentina (Insecta: Odonatoidea) and basic characters states in pterygote wings. *Can. J. Zool.* **62**: 1150–1166 (1984).
9. Desutter-Grandcolas, L. *et al.* 3-D imaging reveals four extraordinary cases of convergent evolution of acoustic communication in crickets and allies (Insecta). *Sci. Rep.* **7**(1) (7099), 1–8 (2017).

**Supplementary movie 1 | *Aeshna isosceles.*** Movie showing the course of the veins, with colors for interpretation (copyright L.J.).

**Supplementary movie 2 | *Aeshna isosceles.*** Movie showing the course of the veins, with colors for interpretation; non-transparent basal part of vein CA (copyright L.J.).

**Supplementary movie 3 | *Aeshna isosceles.*** Movie showing the course of the veins, with colors for interpretation; non-transparent vein CP+ScA (copyright L.J.).

**Supplementary movie 4 | *Aeshna isosceles.*** Movie showing the course of the veins, with colors for interpretation; non-transparent distal part of vein CA (copyright L.J.).

**Supplementary movie 5 | *Aeshna isosceles.*** Movie showing the course of the veins, with colors for interpretation; non-transparent basal part of vein CP (copyright L.J.).

**Supplementary movie 6 | *Aeshna isosceles*.** Movie showing the course of the veins, with colors for interpretation; non-transparent basivenale CB (copyright L.J.).

**Supplementary movie 7 | *Aeshna isosceles*.** Movie showing the course of the veins, with colors for interpretation; non-transparent basal part of vein ScA (copyright L.J.).

**Supplementary movie 8 | *Aeshna isosceles*.** Movie showing the course of the veins, with colors for interpretation; non-transparent basal part of vein ScP (copyright L.J.).

**Supplementary movie 9 | *Aeshna isosceles*.** Movie showing the course of the veins, with colors for interpretation; non-transparent basivenale ScB (copyright L.J.).

**Supplementary movie 10 | *Aeshna isosceles*.** Movie showing the course of the veins, with colors for interpretation; non-transparent Ax0 (copyright L.J.).

**Supplementary movie 11 | *Aeshna isosceles*.** Movie showing the course of the veins, with colors for interpretation; non-transparent vein R and basivenale RB (copyright L.J.).

**Supplementary movie 12 | *Aeshna isosceles*.** Movie showing the course of the veins, with colors for interpretation; non-transparent vein MA (copyright L.J.).

**Supplementary movie 13 | *Aeshna isosceles*.** Movie showing the course of the veins, with colors for interpretation; non-transparent basivenale MBa (copyright L.J.).

**Supplementary movie 14 | *Aeshna isosceles*.** Movie showing the course of the veins, with colors for interpretation; non-transparent vein MP and basivenale MBp (copyright L.J.).

**Supplementary movie 15 | *Aeshna isosceles*.** Movie showing the course of the veins, with colors for interpretation; non-transparent basivenale CuB (copyright L.J.).

**Supplementary movie 16 | *Aeshna isosceles*.** Movie showing the course of the veins, with colors for interpretation; non-transparent basivenale CuBa (copyright L.J.).

**Supplementary movie 17 | *Aeshna isosceles*.** Movie showing the course of the veins, with colors for interpretation; non-transparent basivenale CuBp (copyright L.J.).
